# Self-Control Outdoes Fluid Reasoning in Explaining Vocational and Academic Performance—But Does It?

**DOI:** 10.3389/fpsyg.2020.00757

**Published:** 2020-05-19

**Authors:** Fabian T. C. Schmidt, Christoph Lindner, Julian M. Etzel, Jan Retelsdorf

**Affiliations:** ^1^Department of Educational Psychology, University of Hamburg, Hamburg, Germany; ^2^Leibniz Institute for Science and Mathematics Education, Kiel, Germany

**Keywords:** trait self-control, fluid reasoning, school achievement, standardized tests, interaction effects

## Abstract

Trait self-control, the ability to interrupt undesired behavioral tendencies and to refrain from acting on them, is one of the most important socio-emotional skills. There had been some evidence that it outperforms intelligence in predicting students’ achievement measured as both school grades and standardized achievement tests. However, recent research has shown that the relationships between trait self-control and measures of achievement are more equivocal, emphasizing the importance of the respective outcome of the test to the individual. On the one hand, high-stakes school achievement measures such as GPA repeatedly showed strong relationships with trait self-control. On the other hand, findings on the relationships between trait self-control and performance in mostly low-stakes standardized achievement tests were more heterogeneous. The substantial positive relationship between intelligence and both achievement measures is uncontested. However, the incremental value of trait self-control beyond intelligence when investigating their relationships with achievement remains uncertain. To investigate the relationships of self-control with school achievement and two standardized achievement tests (school mathematics and physics) beyond fluid reasoning, we drew on a large heterogeneous sample of adults in vocational training (*N* = 3,146). Results show differential patterns of results for fluid reasoning and trait self-control and the achievement measures. Trait self-control and fluid reasoning showed similar relationships with school achievement, whereas only fluid reasoning was significantly associated with standardized achievement test scores. For both achievement measures, no significant interaction effects between trait self-control and fluid reasoning were found. The results highlight the utility of trait self-control for performance in high-stakes school assessment beyond fluid reasoning, but set limits to the overall value of trait self-control for achievement in standardized assessments—at least in low-stakes testing situations.

## Introduction

One of the most prominent constructs in research on socio-emotional skills (sometimes called socio-emotional competencies or non-cognitive skills) is trait self-control. It can be defined as the ability to inhibit or overrule immediate urges to attain a long-term goal (De Ridder et al., 2012). However, recent research implies a more resource-oriented conception of trait self-control suggesting that individuals high in trait self-control may use more effortless strategies to exert self-control in addition to impulse inhibition (Gillebaart and De Ridder, 2015).

A broad body of research on the construct cumulated over the past decades, most probably due to its potential relevance for success in school and in the workplace (Gottfredson, 2004; Kuncel et al., 2010). The meta-analysis by De Ridder et al. (2012) showed that having high trait self-control is relevant to a variety of behaviors and outcomes such as happiness and school grades. The authors concluded that the effects of trait self-control are mostly beneficial and adaptive and, thus, that self-control is one of the most beneficial traits in psychology.

The promising findings and the great attention paid to self-control in research, but also in society, led to its consideration in educational policy as one of the most important 21st-century skills (U. S. Department of Education, 2013; UK Department of Education, 2014). However, criticism emerged arguing that the trend toward implementing policies focusing on identifying and fostering socio-emotional skills—such as self-control—and their implementation as relevant constructs in the educational system (e.g., high-stakes character assessment in school) are premature due to a considerable lack of knowledge regarding the utility of these skills (Farrington et al., 2012; Saltman, 2014).

With this study, we aim to contribute to the discussion on the utility of trait self-control, measured using the Brief Self-Control Scale (Tangney et al., 2004). We investigated the incremental validity of trait self-control over fluid reasoning for high-stakes scholastic achievement and low-stakes domain-specific standardized tests. We thereby revisit the notion that trait self-control is a better predictor of school success than intelligence (Duckworth and Seligman, 2005) with a more nuanced design. Recent research indicates that the assumption on the added value of trait self-control over intelligence from earlier studies may have been premature. The relationship may be more complex than previously suggested in so far as that the stakes involved in the achievement outcomes investigated, and the type of the achievement indicators used, can have an impact on the relative importance of trait self-control and intelligence (Bertrams and Dickhäuser, 2009; Lindner et al., 2017; Galla et al., 2019; Lindner and Retelsdorf, 2019). We thus incorporate both standardized tests and grades in our investigation. We argue that these achievement indicators additionally vary in how important they are to the individuals investigated.

## The Trait Self-Control Mode of Action

Trait self-control is considered to be a personality trait that remains relatively stable across situations and time (Gillebaart and De Ridder, 2015). Duckworth and Gross (2014) define trait self-control as the ability to avoid impulsive behavior that enables fulfilling more immediate or short-term obligations. The underlying behavioral mechanism explaining the positive effects of trait self-control has traditionally been assumed to be higher effort investment (Duckworth et al., 2015). Studies found, for example, that individuals with high levels of trait self-control generally invest more personal effort in achievement situations (e.g., Lindner et al., 2018).

The theorizing about how trait self-control affects behavior changed in recent years from focusing on the inability to inhibit impulses to a more resource-oriented approach. Central to this conception is the way in which individuals deal with response conflicts (i.e., competing behavioral tendencies) as introduced by Myrseth and Fishbach (2009). In general, response conflicts arise when a discrepancy exits between activities to reach one’s highly valued overarching goals (e.g., learning to achieve good grades at school to have better opportunities for studying at university) and alternative behaviors that have short-term rewarding values (e.g., watching movies instead of learning for exams). Gillebaart and De Ridder (2015) argue that the success rate of dealing with response conflicts is what distinguishes high and low self-controlled individuals. Individuals with higher self-control are more sensitive to detecting response conflicts, use more effortless strategies to deal with these conflicts, seem to experience these conflicts to a lesser degree, utilize adaptive habitual behavior, and are efficient in downregulating response conflicts before they even become an obstacle. Hence, in contrast to previous assumptions of trait self-control as an effort investment trait (Duckworth et al., 2015), focusing on how individuals with differing levels of trait self-control deal with response conflicts seems promising for understanding the relationships between trait self-control and different achievement outcomes.

It has to be noted that constructs subsumed under the currently popular label socio-emotional skills such as trait self-control or grit tend to fall victim to the jingle and jangle fallacies (e.g., Schmidt et al., 2018). The broad and heterogeneous research on self-control shows similar patterns. We therefore want to be clear that in this study, we explicitly use the term *trait self-control* to refer to the personality trait in the conscientiousness domain and, thus, do not incorporate other relevant topics in psychological research such as metacognitive strategies (Zimmerman and Kitsantas, 2005) or state self-control capacity (Lindner et al., 2019). On a theoretical level, trait self-control and conscientiousness are closely related. Roberts et al. (2005) argue that trait self-control can best be viewed as a lower-order facet in their hierarchical conscientiousness model. The relevance of conscientiousness for scholastic achievement and achievement later in life is uncontested (Poropat, 2009; Spengler et al., 2014, 2015). Researchers found similar results to the findings presented by Duckworth and Seligman (2005) with regard to the incremental relationship of conscientiousness over intelligence for scholastic achievement (e.g., Barton et al., 1972; Chamorro-Premuzic and Furnham, 2008; Furnham and Monsen, 2009; Spengler et al., 2016). With the present investigation, we aimed, on the one hand, to expand on the findings by Duckworth and Seligman (2005). On the other hand we wanted to shed light on trait self-control, as a popular construct among the socio-emotional skills. This approach is in line with the recent trend toward investigating facets in contrast to broad domains (Mõttus et al., 2017; Schmidt et al., 2020). However, we included a measure to assess conscientiousness to broaden the perspective of the presented research.

## Trait Self-Control and Academic Achievement

Research shows that trait self-control is an important predictor of students’ achievement-related learning behavior (e.g., Zimmerman and Kitsantas, 2005) at school (e.g., Bertrams and Dickhäuser, 2009), in university (e.g., Tangney et al., 2004), and in vocational education and training (Lindner et al., 2015). Researchers explain this relationship on different levels. Duckworth et al. (2012) claim that being self-controlled is advantageous in school when studying the contents of what is formally taught, leading to an increase in GPA through higher-valued learning outcomes and, in addition, through behavior in the classroom that may be factored into report card grades by teachers directly (Brookhart, 1994; Cizek et al., 1995; McMillan et al., 2002). In a similar vein, findings show that more self-controlled individuals behave better in the classroom (Valiente et al., 2008), show better completion of sometimes strenuous homework assignments, and show overall more effortful behavior in school (Duckworth and Seligman, 2005).

Up until now, studies only rarely recognized the differences in the relevance of the achievement measures that were used. We assume that the influence of trait self-control on an achievement outcome varies with the subjective importance an individual subscribes to that very outcome. Derived from the considerations on the differential impact of trait self-control on the perception and handling of response conflicts in high-stakes and low-stakes situations, it seems essential to address this issue in trait self-control research. In contrast to low-stakes standardized assessments for the purposes of research, school grades are of great importance for the start of work life or post-compulsory education (Brookhart, 1991). Hence, the perception of response conflicts may partly explain the stronger positive relationships between trait self-control (i.e., defined as a trait that enables individuals to sensitively detect and handle response conflicts) and personal highly valued grades. On the other hand, the findings on the relationship with achievement outcomes in standardized tests may be explained by the absence of response conflicts (i.e., test results have no personal consequences for individuals’ future).

## Trait Self-Control and Standardized Achievement Tests

Studies that investigated the impact of trait self-control when individuals are required to invest effort in order to solve items in standardized achievement tests are scarcer than studies focusing on the relationship between trait self-control and GPA. Studies investigating the relationships with domain-specific achievement tests—especially tests that aim to assess curriculum-derived competencies relevant to the tested individuals—are scarce as well.

The findings on the relationships between trait self-control and achievement in standardized achievement tests vary to a certain degree. Whereas some studies found positive relationships between trait self-control and achievement in standardized tests (Duckworth and Seligman, 2005; Bertrams and Dickhäuser, 2009), other studies did not find a significant relationship between trait self-control and achievement (Lindner et al., 2017; Lindner and Retelsdorf, 2019). Interestingly, achievement in a mathematics tests showed positive relationships with trait self-control when the students were graded for their performance in the test (Bertrams, 2012). These findings imply that the importance of the outcome of a standardized test has an impact on the way trait self-control interacts with the way the test is completed.

Hence, response conflicts may play a role in the way that the importance of the consequences of the test results may influence the tested individual. The effects response conflicts can have on the achievement in standardized tests in low-stakes situations may be negligible and vice versa for high-stakes situations. Gillebaart and De Ridder (2015) argue that trait self-control is associated with a higher sensitivity for detecting response conflicts. Since no or only small response conflicts need to be overcome in low-stakes assessment, the impact of trait self-control on the performance in achievement tests should be limited. The effortless self-control strategies that highly self-controlled individuals possess would not necessarily be utilized due to the non-existent response conflicts and thus, in theory, have no impact on the low-stakes assessment performance. In high-stakes achievement situations, individuals have to prepare themselves for reaching highly valued overarching goals (e.g., getting good grades in the upcoming exam) instead of following more rewarding and less effortful activities (i.e., watching movies). Therefore, trait self-control is required to overcome such response conflicts.

However, findings also support the notion of trait self-control defined as an effort investment trait (Duckworth et al., 2015). Studies, for example, found that more self-controlled students show more perseverance in time-consuming, controlled information processing when working on standardized low-stakes achievement tests (Lindner et al., 2018). In another study, trait self-control has been found to stand in positive relationship to the amount of effort and time-on-task invested in an achievement test in mathematics (Lindner et al., 2017), whereas no relations were found between trait self-control and test performance in mathematics. Similar results were found in another study by Lindner and Retelsdorf (2019), who investigated the relations between trait self-control and achievement-related outcomes (test taking effort, motivation, and performance) in a low-stakes test for assessing English as a foreign langue. All in all, the presented findings indicate support for both approaches to trait self-control. With this study, we aim to shed light on the degree to which one or the other approach may be more suitable to explain the differential relationships between trait self-control and high-stakes and low-stakes achievement indicators.

## Relative Influences of Trait Self-Control and Intelligence on Achievement

Duckworth et al. (2012) showed that school grades stand in closer relationship to trait self-control than to intelligence, whereas standardized achievement stands in closer relationship to intelligence than to trait self-control. The authors argue that these differences in relationships reflect the differing competencies assessed in GPA versus standardized tests. Grades in school are influenced not only by the teachers’ assessment of the contents the students actually learned but, in addition, by the behavior inside the classroom (e.g., participation or attendance) and outside the classroom (e.g., homework completion). School grades thus represent an amalgamation of multiple factors that are influenced by the assessment of curricular competence *and* scholastic behaviors, which are in turn influenced by socio-emotional skills (Farrington et al., 2012). A recent study by Galla et al. (2019) showed not only that school grades are better than high-stakes admission test scores in predicting on-time college graduation but also that 40% of the variance of the grades can be explained by measures of self-regulation in contrast to only 3% of the high-stakes SAT scores. These findings can be explained by the teachers’ explicit and implicit inclusion of socio-emotional skills in their grading process. Furthermore, and central to the present investigation, school grades are highly important achievement outcomes for the individual (Galla et al., 2019). More self-controlled individuals are held to be better prepared to perceive and tackle response conflicts that arise in high-stakes situations (Gillebaart and De Ridder, 2015). Hence, trait self-control factors into the grading process in more than one way.

With the application of standardized achievement tests, policy makers, administrators, and researchers alike aim to acquire a purer reflection of competence rather than to test for the competence acquired of the curricula that students were actually exposed to. In addition, the research on the relationship between standardized tests and school achievement—or trait self-control for that matter—mostly uses composite measures of a broad set of competencies. Thus, school grades and standardized achievement tests differ in not only the competencies they aim to assess but to what end they are administered. In addition, standardized tests for the purposes of research mostly represent low-stakes testing situations. It appears obvious that intelligence can be expected to show stronger relationships with standardized achievement tests, as both refer specifically to the performance on a set of cognitive tests directly (as intended by tests of cognitive ability) or indirectly (through testing a broad set of competencies that are not necessarily part of the curriculum the students came in contact with). The findings by Duckworth et al. (2012) confirm these assumptions. Notably, it cannot be ruled out that these results to a degree stemmed from shared method variance (mono-method-bias) to the degree that the strength of relationships is in part a result of similar methods used.

To circumvent this problem to a certain extent, we used a domain-specific standardized low-stakes achievement test that reflects a curriculum-bound assessment of competencies relevant to the individuals in the vocational training. To the best of our knowledge, no empirical studies have analyzed the relationship between school grades and domain-specific tests on the one hand and trait self-control as well as fluid reasoning on the other hand, with the exception of Duckworth et al. (2012).

## The Present Investigation

Is trait self-control more strongly associated with achievement than fluid reasoning, or does it only stand out in situations that are more important to the individual as recent conceptual changes in trait self-control research imply? To address these issues, we used a large and heterogeneous sample of young adults at the beginning of their vocational training and investigated the incremental validity of trait-self-control over fluid reasoning for not only school achievement but also domain-specific standardized achievement test scores (mathematics and physics) that reflect relevant domains of competence for the individuals in the sample. In line with the novel conceptualization of trait self-control, we would further argue that school achievement could be conceptualized as high-stakes and the standardized tests as lower-stakes, a conceptualization we discuss down below. Our research thus enables us to get a better understanding of the socio-emotional skill as well as the differential relationships between trait self-control and two relevant indicators of achievement. Our research also contributes to the ongoing discussion on the utility of the social–emotional skill.

Derived from the theoretical assumptions with regard to trait self-control and response conflicts, we would assume that trait self-control is more important for high-stakes scholastic achievement than the lower-stakes achievement in the standardized tests. We would assume to find positive relationships between fluid reasoning and school achievement and even stronger relationships with the standardized tests, in part due to the higher methodological similarity. Derived from earlier research on the added value of trait self-control over intelligence, we assume trait self-control to be at least as important as fluid reasoning with regard to grades. In contrast, we do not hypothesize that trait self-control outdoes fluid reasoning with regard to the standardized tests in mathematics and physics, even though the tests are domain-specific, and thus, the methodological similarity can be assumed to be less relevant than in earlier studies that used standardized assessments of broad school achievement.

In addition, we assume that individuals higher in fluid reasoning may profit more from being more self-controlled. Following the argumentation by Gillebaart and De Ridder (2015), individuals high in trait self-control handle response conflicts more advantageously. Subsequently, they should profit more from better fluid reasoning. Thus, we assume to find a positive interaction effect for high-stakes school achievement. For the low-stakes achievement tests, we were not able to derive a concrete hypothesis from the scarce literature on the topic. Therefore, we keep the investigation of the interaction effect on the standardized tests exploratory in nature. However, it seems less plausible to find an interaction effect for the low-stakes testing situation, as we would assume to have less pronounced response conflicts.

## Materials and Methods

### Sample

The data stemmed from the study Mathematics and Science Competencies in Vocational Education and Training (ManKobE; cf. Retelsdorf et al., 2013). The sample consisted of trainees in different vocational fields, namely industrial clerks and technicians, with the latter consisting of car mechatronics, industrial, and electrical technicians as well as chemical and biological laboratory assistants. The final sample comprised *N* = 3,146 trainees. Participants’ average age was 18.58 (*SD* = 2.77), and 38.5% of the participants were female. In the sample, 20% reported having at least one parent born outside of Germany. The data were assessed in five German federal states (Bavaria, Hesse, Lower Saxony, North Rhine-Westphalia, and Baden-Württemberg).

This study was carried out in accord with the ethical guidelines for research with human participants as proposed by the American Psychological Association (APA). The study materials and procedures were approved by the Ministries of Education and Cultural Affairs of the Federal States of Hesse, Bavaria, North Rhine-Westphalia, Lower Saxony, and Baden-Württemberg. The data were collected by qualified research assistants under the administration of the Data Processing and Research Center in Hamburg, which is part of the International Association for the Evaluation of Educational Achievement (IEA). Before data collection, the Data Processing Center in Hamburg obtained written informed consent from all participants and—if not of legal age—their parents. The analysis scripts of our reported results, the relevant data to reproduce these results, and a list of publications using data from the ManKobE project are open and available to download (Schmidt, 2020).

### Measures

#### Trait Self-Control

Trait self-control was assessed using the adapted German version (α = 0.82; Bertrams and Dickhäuser, 2009) of the Brief Self-Control Scale (Tangney et al., 2004). All 13 items (e.g., “I say inappropriate things.”) were rated on a five-point Likert scale, anchored at 1 “not at all like me” and 5 “very much like me.”

#### Conscientiousness

We assessed conscientiousness with the Big Five Inventory-2 (BFI-2; α = 0.76; Soto and John, 2017; German version: Lang et al., 2001). The BFI-2 facets were constructed to strike a balance between bandwidth and fidelity using 12 items to assess the personality trait (e.g., “I am someone who is systematic, likes to keep things in order”). The same five-point Likert-type scale as for trait self-control was used as the response format.

#### Fluid Reasoning

Domain-general fluid reasoning was assessed by three subtests of the Cognitive Ability Test (Heller and Perleth, 2000). These subtests examine reasoning in the verbal (20 items), numerical (20 items), and figural (25 items) domains. In the present study, weighted likelihood estimates (WLEs; WLE reliability = 0.90) from a composite one-dimensional model were used as individual scores for further analyses. Reasoning subtests are considered a fair indicator of general intelligence (Neisser et al., 1996).

#### Mathematics and Physics Achievement

We assessed mathematics and physics achievement with tests developed by the Institute for Educational Quality Improvement in Berlin. The tests are based on the German Educational Standards in mathematics and physics (Pant et al., 2013) and thus assess curriculum-derived proficiency in the two domains, which are important in vocational training. The tests were administered using a matrix design in which trainees worked only on a subset of the items (mathematics, 34 items, and physics, 40 items). Again, individual scores were calculated in the form of WLEs with acceptable reliabilities of 0.65 (mathematics) and 0.67 (physics) due to the heterogeneity of the competencies measured.

#### Major GPA

The major GPA (mGPA) is an aggregate of the grades in the first and second languages as well as the grades in mathematics and the compulsory optional subjects. In our study, we used the grades in the main subjects that are compulsory and the grades in the optional subjects that the students are required to take (but can choose from a set of subjects). The German grading system ranges from 1 (outstanding) to 6 (fail). To facilitate the interpretation of our results, school grades were reverse-coded so that higher scores reflected more positive outcomes.

#### Control Measures

Trainees’ socioeconomic status was indicated by the highest parental score (either mother or father) on the International Socio-Economic Index of Occupational Status (HISEI; Ganzeboom et al., 1992). Because large variances lead to convergence problems, the HISEI was divided by 100. This transformation only affects the variable’s raw metric and has no influence on the standardized results reported below. Migrant status was dummy-coded (0 = both parents born in Germany, 1 = one or both parents born outside Germany). Finally, participants’ age and gender (female = 1, male = 2) were used in the present study.

### Analyses

All multiple regressions were estimated in Mplus, Version 8.1 (Muthén and Muthén, 1998). Because students were clustered in vocational school classes, we accounted for potential dependencies by obtaining cluster robust standard errors via the Mplus option “TYPE = COMPLEX.” We probed all interactions using the Johnson–Neyman method (Johnson and Neyman, 1936; Hayes, 2013) to identify regions of significance. All dependent variables (mGPA, mathematics achievement, and physics achievement) were estimated simultaneously (see Appendix Table A1 for the stepwise regression for trait self-control, conscientiousness, and fluid intelligence). We controlled for the effects of gender, age, HISEI migration status, and conscientiousness in our analyses. Missing data were handled via full information maximum likelihood estimation accounting for missing data (on average, 11.4% of the data were missing). We estimated the models again using listwise deletion to obtain an indicator of the robustness of the results. The resulting relationship patterns were virtually identical to those reported below (see Schmidt, 2020, to find the analysis scripts and results). Moreover, no *a priori* analysis of statistical power was conducted. However, a *post hoc* power analysis for the final model with a small effect size reveals a power of 1-β = 0.99 using the given sample size with GPower (Faul et al., 2007).

## Results

The correlations between all dependent and independent variables as well as all control measures used in the study can be found in Table 1. As expected, fluid reasoning as assessed in our study showed strong relationships with the standardized test results for mathematics and physics and a weaker but statistically significant correlation with mGPA. Trait self-control, on the other hand, showed the expected strong relationships with mGPA but no statistically significant relationships with either standardized achievement test. The relationship between trait self-control and conscientiousness was substantial (shared variance 34%).

**TABLE 1:** Descriptive statics and observed correlations of study variables.

	*M*	*SD*	1	2	3	4	5	6	7	8	9	10
(1) Gender			–									
(2) Age	18.58	2.77	−0.08*	–								
(3) Migration status			0.08*	0.11*	–							
(4) HISEI	48.69	18.16	−0.05*	0.06*	−0.21*	–						
(5) mGPA	4.44	0.58	−0.22*	−0.15*	−0.09*	0.03	–					
(6) Math score	0.81	1.31	−0.05*	0.07*	−0.16*	0.11*	0.13*	(0.65)				
(7) Physics score	0.79	1.17	−0.08*	0.10*	−0.21*	0.17*	0.14*	0.49*	(0.67)			
(8) Fluid rea.	0.05	0.97	−0.21*	0.17*	−0.19*	0.14*	0.14*	0.53*	0.60*	(0.90)		
(9) TSC	3.27	0.62	−0.12*	0.03	−0.04*	–0.03	0.16*	0.03	0.01	0.05*	(0.81)	
(10) Conscientiousness	3.56	0.47	−0.18*	0.10*	−0.04*	–0.01	0.17*	0.06*	0.05*	0.08*	0.58*	(0.76)

TSC, trait self-control; HISEI, Highest International Socio-Economic Index of Occupational Status; mGPA, major grade point average; fluid rea., fluid reasoning. Reliabilities in parentheses. *p < 0.05.

To test for the incremental validity of trait self-control over fluid reasoning as assessed in our study, we estimated a simultaneous multiple regression with the covariates named earlier. The regression coefficients of the standardized solution from the regression model with trait self-control, fluid reasoning, and the interaction between the two can be found in Table 2. For all dependent measures, significant associations for fluid reasoning emerged. On average, higher trait self-control was associated with higher mGPA but not with higher test scores in mathematics and physics. Combined, the constructs explained substantially more variance of the achievement tests (math, 29%; physics, 37%) than they did for mGPA (12%). The inclusion of conscientiousness did not change the amount of explained variance for all three outcomes (see Appendix Table A1 for the results from a stepwise regression approach). The results thus indicate that for mGPA, conscientiousness and trait self-control are equally important predictors, even though conscientiousness overall showed stronger relationships with mGPA than trait self-control in the final model (see Table 2).

**TABLE 2:** Standardized slope estimates of the multivariate regression analyses.

	mGPA			Mathematics test score			Physics test score		
	Est.	95% CI	*p*	Est.	95% CI	*p*	Est.	95% CI	*p*
Gender	–0.17	[−0.21, −0.13]	0.000	0.07	[0.03, 0.10]	0.000	0.05	[0.01, 0.08]	0.009
Age	–0.23	[−0.28, −0.18]	0.000	–0.01	[−0.05, 0.03]	0.625	0.01	[−0.02, 0.04]	0.589
HISEI	0.01	[−0.03, 0.05]	0.539	0.03	[0.00, 0.07]	0.036	0.07	[0.04, 0.11]	0.000
Migration Status	–0.02	[−0.06, 0.03]	0.408	–0.06	[−0.10, −0.03]	0.000	–0.09	[−0.12, −0.06]	0.000
BFI-2 Con.	0.11	[0.06, 0.15]	0.000	0.03	[−0.01, −0.07]	0.122	0.03	[−0.01, 0.06]	0.161
TSC	0.07	[0.02, 0.11]	0.008	–0.01	[−0.05, 0.03]	0.710	–0.02	[−0.06, 0.01]	0.187
Fluid Rea.	0.12	[0.07, 0.17]	0.000	0.52	[0.48, 0.56]	0.000	0.58	[0.55, 0.61]	0.000
TSC x Fluid Rea.	0.03	[−0.01, 0.07]	0.134	0.00	[−0.03, 0.04]	0.822	0.01	[−0.02, 0.04]	0.460
	*R*^2^ = 0.12 (adjusted *R*^2^ = 0.12)			*R*^2^ = 0.29 (adjusted *R*^2^ = 0.29)			*R*^2^ = 0.37 (adjusted *R*^2^ = 0.37)		

*TSC, BSCSTrait Self-control; HISEI, Highest International Socio-Economic Index of Occupational Status; BFI-2 Con., BFI-2 Conscientiousness; mGPA, major grade point average; est., estimate; CI, confidence interval; fluid rea., fluid reasoning.*

In line with previous research, fluid reasoning proved to be relatively more important for achievement in standardized tests; the coefficients for fluid reasoning were overall larger than those for trait self-control. For mGPA, on the other hand, trait self-control emerged to be equally important. For all three indicators of achievement, no significant interaction effects (fluid reasoning × trait self-control) emerged.

**FIGURE 1:**
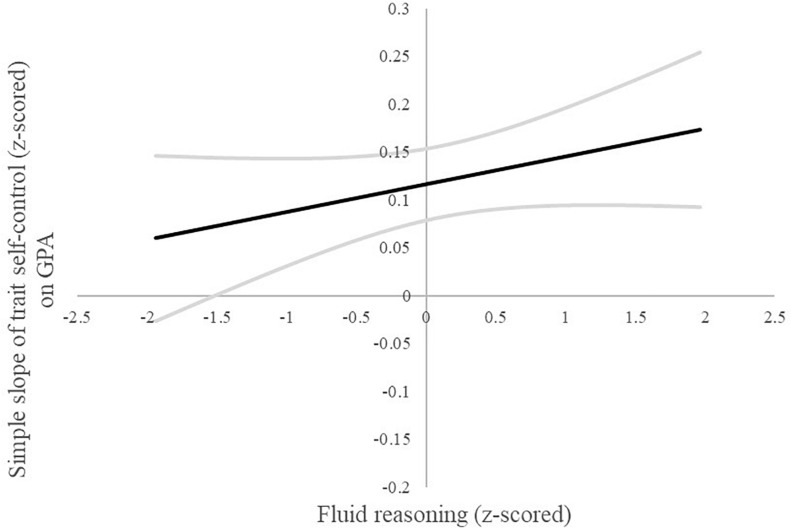
Johnson–Neyman plot of the simple slope (95% upper and lower limit grayed out) of trait self-control on GPA across the range of fluid reasoning (±2 SD).

**FIGURE 2:**
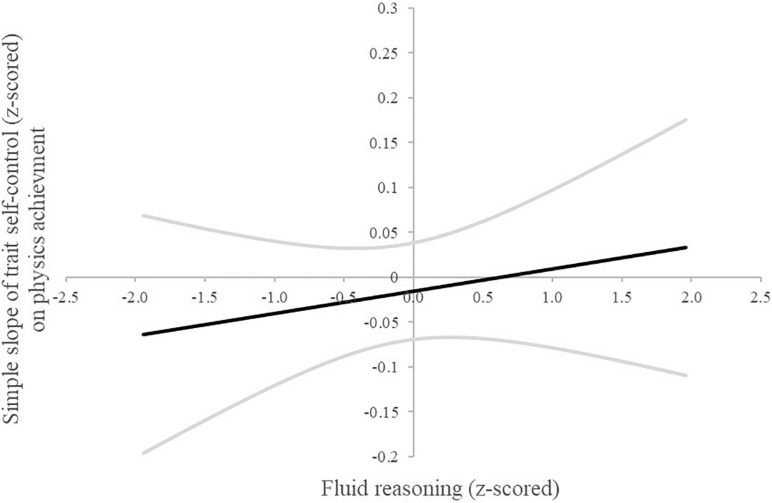
Johnson–Neyman plot of the simple slope (95% upper and lower limit grayed out) of trait self-control on physics achievement across the range of fluid reasoning (±2 SD).

**FIGURE 3:**
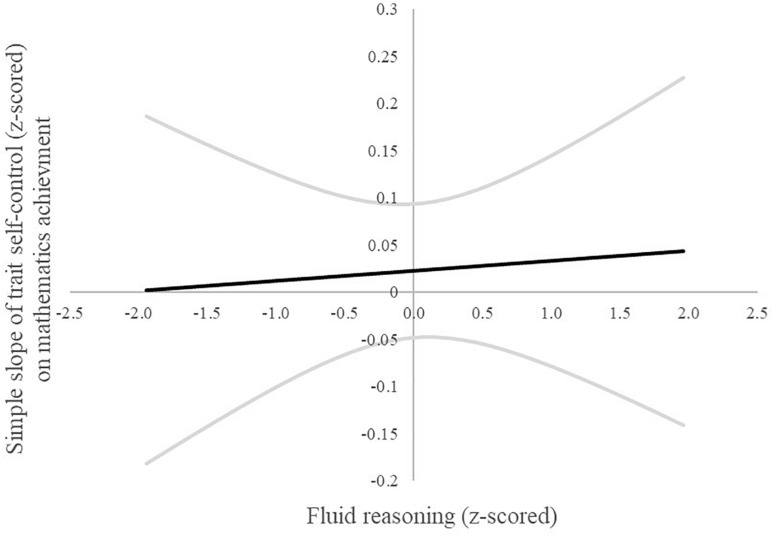
Johnson–Neyman plot of the simple slope (95% upper and lower limit grayed out) of trait self-control on mathematics achievement across the range of fluid reasoning (±2 SD).

A follow-up Johnson–Neyman procedure for plotting interactions (Johnson and Neyman, 1936; Hayes, 2013) can reveal regions of significance even if the overall interaction effect is non-significant or small, as they typically are (Nagengast et al., 2011). The Johnson–Neyman procedure revealed that the association of trait self-control with mGPA was significantly positive among individuals scoring higher than −1.5 *SD* below average on the fluid reasoning measure (see Figure 1). These findings imply that the individuals higher in fluid reasoning profit with regard to scholastic achievement by being more self-controlled. In other words, apart from the individuals scoring on the very low end on the fluid reasoning scale, individuals higher in fluid reasoning profit more from higher scores in trait self-control. No such interactions were found in the follow-up Johnson–Neyman analysis for physics (Figure 2) and mathematics (Figure 3) achievement.

## Discussion

Socio-emotional skills have become popular among researchers as well as practitioners and politicians, as they offer a new perspective on pathways to achievement in education and in the workplace. To replace cognitive skills to a degree with socio-emotional skills as crucial prerequisites for achievement is a prominent idea not only due to the notion of their potential higher malleability but also because these constructs have been offered as a potential gateway to more equal opportunity to success (Farrington et al., 2012). However, critics pointed to a number of unresolved issues and questioned the *de facto* utility of the so-called socio-emotional skills. Which of the current conceptualizations of trait self-control is more suitable to explain achievement? Is trait self-control superior to intelligence in explaining high-stakes teacher assessment of proficiency as measured by grades? Does trait self-control show stronger associations with achievement in curriculum-based lower-stakes standardized achievement tests than intelligence?

Trait self-control is one of the most prominent socio-emotional skills that attracted attention based on the promise to explain achievement above intelligence. The conceptualization of the construct trait self-control, however, changed in the recent years, and open questions on the underlying mechanisms of the construct emerged. Research showed it to stand in positive relationship, for example, with school performance, income, and employment (De Ridder et al., 2012; Duckworth and Carlson, 2013). Duckworth et al. (2015) explained these findings with the higher effort individuals invest in pursuing their goals in addition to the better inhibition of falling victim to alluring alternative behavioral tendencies. Recent research brings the importance of the consequences of the tests taken into focus. Gillebaart and De Ridder (2015) argue that highly self-controlled individuals are more able to avoid and regulate response conflicts and therefore achieve their pursued goals more successfully.

With the present study, we aimed to add to the ongoing debate on the conceptualization and utility of trait self-control and investigated the associations of trait self-control with high-stakes school achievement as measured by mGPA and two low-stakes standardized achievement tests beyond fluid reasoning using a broad and heterogeneous sample. The utility of fluid reasoning outweighs trait self-control for the domain-specific standardized achievement tests in mathematics and physics. Other than in previous studies (e.g., Duckworth and Seligman, 2005), the achievement tests were not domain-general measures of broad scholastic proficiency. We used tests to assess mathematics and physics achievement that were developed with the aim to assess curriculum-relevant achievement. Thus, the relevance of fluid reasoning is uncontested, and trait self-control does not add when comparing these constructs head-to-head.

Trait self-control did significantly show associations with mGPA over fluid reasoning (and conscientiousness) but, in contrast to earlier findings, did not surpass fluid reasoning, restricting its relevance as a socio-emotional skill to a degree. However, the results of our study hint on the relevance of response conflict resolution ability of more self-controlled individuals (Gillebaart and De Ridder, 2015) rather than the effort investment hypothesis (Duckworth et al., 2015). We were able to show that trait self-control plays a role in school achievement as measured by grades as opposed to achievement in the low-stakes standardized tests. We can only assume that the significant relationships between trait self-control and mGPA result at least partly from the higher stakes involved and the resulting more effortless strategies to show self-controlled behavior. In situations in which response conflicts occur (high-stakes situations), trait self-control seems to give individuals the edge in showing their true potential, whereas in low-stakes situations, trait self-control seems to be less relevant. Notably, the associations between trait self-control and mGPA remained statistically significant when controlling for conscientiousness, even though the relationship was weaker after conscientiousness was introduced. Trait self-control can be seen as a facet in the conscientiousness domain (Roberts et al., 2005). Thus, our results are in line with the reasoning by Mõttus et al. (2017), who argues that it can be worthwhile to investigate lower-order facets such as trait self-control. However, our results show that conscientiousness in part outperformed trait self-control, as it showed overall slightly stronger associations with mGPA.

Even though the results of this study imply that the stakes involved stand in relationship with the impact trait self-control can have on achievement, and thus serve as an argument for the importance of response conflicts, this study cannot explain if the response conflict resolution is just a preliminary step in achieving valued goals and the subsequent higher-effort investment actually explains the results. Furthermore, the results by Galla et al. (2019) show the substantial overlap between grades and measures of self-regulatory competencies, indicating common method variance that may exaggerate the differential findings to a degree. In a similar vein, Duckworth et al. (2012) argue that self-control shows differing relationships with GPA and standardized tests because these indicators reflect different competencies. It has to be noted that in contrast to the study by Duckworth et al. (2012), the standardized tests we used are more ecologically valid. In addition, it is still unclear to what degree the higher social acceptance of the academic behavior or, for example, the more habitualized learning behavior the more self-controlled students show impacts the grading of the teachers or if the behavior shown in class may even be more or less independent from personality factors (Spengler et al., 2018). It thus seems worthwhile to investigate the factors influencing the grading process more closely in future research. In addition, our findings on the differential relationship between fluid reasoning and trait self-control and mGPA may in part be explained by the more heterogeneous sample we used. Most previous studies investigating the incremental validity of trait self-control over intelligence for academic achievement used highly selected samples of university students for which a restriction in the variance of intelligence can be expected. This may result in an unwanted deflation of the associations between intelligence and achievement and in turn lead to an overestimation of the association between academic achievement and trait self-control in comparison to intelligence.

Fluid reasoning and trait self-control did not interact statistically significantly; however, the *post hoc* Johnson–Neyman analysis revealed some interesting information on the associations with mGPA. They suggest that students higher in fluid reasoning may profit more from higher scores in trait self-control. Only the students on the very low end of the fluid reasoning spectrum in our sample did not profit significantly from higher scores in trait self-control. These findings to a degree question the usefulness of efforts to foster trait self-control in students in need as suggested by the policy decisions named earlier (U. S. Department of Education, 2013; UK Department of Education, 2014), as they imply that students would not benefit equally from these endeavors. Further research should consider taking a closer look at the interplay between socio-emotional skills, intelligence, and other relevant factors for success when investigating their impact on academic achievement to determine if our *post hoc* analyses are in fact meaningful. In addition, the consideration of facets is useful not only with regard to non-cognitive personality traits as mentioned above but also with regard to cognitive personality traits (Kretzschmar et al., 2018). An investigation on the facet level of intelligence would therefore be a welcomed addition to the literature.

All in all, our results highlight the utility of trait self-control, as it shows significant relationships with broader measures of high-stakes school performance to a degree, but set limits to more objective and lower-stakes assessments of achievement. The findings thus replicate the study by Duckworth et al. (2012) only in part, as we did not find trait self-control to show substantially higher associations to achievement than fluid reasoning. However, our results are in accordance with the more recent conceptualization of trait self-control emphasizing response sensitivity rather than effort investment to explain the association of trait self-control with achievement.

### Limitations and Future Directions

Finally, some limitations of the present study need to be addressed. First, our samples only comprised students from vocational training, limiting the generalizability of the findings. Second, we were only able to use cross-sectional data; longitudinal surveys are needed to confirm the findings. Third, we only used self-report measures to assess trait self-control, the limits of which are well documented (Lucas and Baird, 2006). Furthermore, we did not correct for measurement error in our analyses. The results thus may represent a conservative estimation of the actual effect sizes. Finally, the measure we used to assess school achievement (mGPA) should not be mistaken for the widely used GPA. The mGPA consists of the compulsory subjects including optional subjects. Thus, the mGPA is a less broad measure of scholastic achievement than the GPA. This is a limitation that needs to be kept in mind when interpreting our results, such as the comparatively weak relationship between mGPA and fluid reasoning and the overall lower percentage of variance explained in mGPA in the regression analyses.

It must be noted that the explanatory power of our findings is limited to the degree that common method variance may have influenced the results (for a discussion, see Lechner et al., 2017). In an earlier study, Duckworth and Seligman (2005) similarly suspected that the common variance between intelligence and the achievement test score was due to shared method variance. They went on to argue that independent from actual knowledge or ability, some students may perform well in multiple-choice items under time constraints regardless of their content. However, in the present investigation, we decided to include domain-specific tests that were developed to assess curriculum-relevant content for adults in vocational training. We therefore would assume that the effect the common method variance has on our results may in fact at least be smaller than in the previous studies. In addition, the results with regard to the differential associations of fluid reasoning and trait self-control with achievement in high-stakes and low-stakes situations cannot be compared head-to-head with the data we used. A more elaborate approach would be to find more similar indicators of achievement or competence in high-stakes and low-stakes situations that would make it possible to, for example, investigate foreign language competence in high-stakes and low-stakes situations in parallel. Such a design would enable giving a better indication on the impact of fluid reasoning and trait self-control with regard to the relevance of the outcome of the test.

Furthermore, it has to be noted that we used a domain-general operationalization of fluid reasoning. Previous research showed that different facets of intelligence can lead to differing relationships with personality traits such conscientiousness (Kretzschmar et al., 2018). The results of our investigation might change considerably when investigating the facets separately; thus, our results need to be interpreted with this restriction in mind. We would encourage further research in this domain (e.g. Schmidt et al., 2019).

Finally, we cannot be certain that our presumption on the subjective perception of high-stakes and low-stakes situations is correct. We can only assume that the subjective relevance of mGPA is higher to the individuals in our sample than the results of the standardized tests. The tests were not used to give feedback to the individuals in vocational training, nor were the individual results submitted to the teachers or other stakeholders. The students were reimbursed for their participation but were not specifically incentivized for higher achievement or higher effort. Nevertheless, studies investigating the impact of the stakes involved in a testing situation on trait self-control should preferably include explicit measures to assess the relevance of the testing situations. Such approaches would enable getting a more in-depth grasp on the mechanisms behind trait self-control, for example, whether there exists a differential or even a combined sequential impact of response sensitivity and effort investment on achievement.

## Data Availability Statement

The datasets generated for this study are available on request to the corresponding author. The analysis scripts of our reported results, the relevant data to reproduce these results, and a list of publications using data from the ManKobE project are open and available to download (Schmidt, 2020).

## Ethics Statement

The studies involving human participants were reviewed and approved by Ministries of Education and Cultural Affairs of the Federal States of Bavaria, Hesse, Lower Saxony, North Rhine-Westphalia, and Baden-Württemberg. Written informed consent to participate in this study was provided by the participants’ legal guardian/next of kin.

## Author Contributions

FS: conceptualization, formal analysis, writing original draft, and editing. CL: conceptualization, editing, and review. JE: data curation, formal analysis, and methodology. JR: conceptualization, editing, and review.

## Conflict of Interest

The authors declare that the research was conducted in the absence of any commercial or financial relationships that could be construed as a potential conflict of interest.

## Appendix

**TABLE A1:** Standardized slope estimates of the multivariate regression analyses.

	M1			M2			M3			M4		
	Est.	95% CI	*p*	Est.	95% CI	*p*	Est.	95% CI	*p*	Est.	95% CI	*p*
*Outcome: mGPA*												
Gender	–0.22	[−0.26, −0.18]	0.000	–0.18	[−0.22, −0.14]	0.000	–0.18	[−0.22, −0.14]	0.000	–0.17	[−0.21, −0.13]	0.000
Age	–0.20	[−0.24, −0.15]	0.000	–0.22	[−0.27, −0.17]	0.000	–0.23	[−0.28, −0.18]	0.000	–0.23	[−0.28, −0.18]	0.000
HISEI	0.02	[−0.02, 0.05]	0.431	0.01	[−0.03, 0.05]	0.604	0.01	[−0.03, 0.05]	0.596	0.01	[−0.03, 0.05]	0.539
Migration Status	–0.05	[−0.09, 0.00]	0.027	–0.02	[−0.06, 0.02]	0.366	–0.02	[−0.06, 0.02]	0.361	–0.02	[−0.06, 0.03]	0.408
BFI-2 Con.	–			–			0.14	[0.10, 0.18]	0.000	0.11	[0.06, 0.15]	0.000
TSC	–			0.13	[0.08, 0.16]	0.000	–			0.07	[0.02, 0.11]	0.008
Fluid Rea.	–			0.12	[0.07, 0.17]	0.000	0.12	[0.07, 0.17]	0.000	0.12	[0.07, 0.17]	0.000
TSC x Fluid Rea.	–			0.03	[−0.01, 0.07]	0.139	–			0.03	[−0.01, 0.07]	0.134
	*R*^2^ = 0.09 (adjusted *R*^2^ = 0.09)			*R*^2^ = 0.12 (adjusted *R*^2^ = 0.12)			*R*^2^ = 0.12 (adjusted *R*^2^ = 0.12)			*R*^2^ = 0.12 (adjusted *R*^2^ = 0.12)		
*Outcome: Mathematics Test Score*												
Gender	–0.02	[−0.07, 0.02]	0.257	0.07	[0.03, 0.10]	0.000	0.07	[0.03, 0.10]	0.000	0.07	[0.03, 0.10]	0.000
Age	0.09	[0.05, 0.13]	0.000	–0.01	[−0.05, 0.03]	0.709	–0.01	[−0.05, 0.03]	0.633	–0.01	[−0.05, 0.03]	0.625
HISEI	0.08	[0.04, 0.12]	0.000	0.03	[0.00, 0.06]	0.037	0.03	[0.00, 0.06]	0.035	0.03	[0.00, 0.07]	0.036
Migration Status	–0.15	[−0.19, −0.12]	0.000	–0.06	[−0.10, −0.03]	0.000	–0.06	[−0.10, −0.03]	0.000	–0.06	[−0.10, −0.03]	0.000
BFI-2 Con.	–			–			0.03	[−0.01, 0.06]	0.117	0.03	[−0.01, −0.07]	0.122
TSC	–			0.01	[−0.02, 0.04]	0.522	–			–0.01	[−0.05, 0.03]	0.710
Fluid Rea.	–			0.52	[−0.48, 0.56]	0.000	0.52	[0.48, 0.56]	0.000	0.52	[0.48, 0.56]	0.000
TSC x Fluid Rea.	–			0.00	[−0.03, 0.04]	0.814	–			0.00	[−0.03, 0.04]	0.822
	*R*^2^ = 0.04 (adjusted *R*^2^ = 0.04)			*R*^2^ = 0.29 (adjusted *R*^2^ = 0.29)			*R*^2^ = 0.29 (adjusted *R*^2^ = 0.29)			*R*^2^ = 0.29 (adjusted *R*^2^ = 0.29)		
*Outcome: Physics Test Score*												
Gender	–0.06	[−0.10, −0.01]	0.013	–0.04	[0.01, 0.08]	0.014	–0.04	[0.01, 0.08]	0.009	0.05	[0.01, 0.08]	0.009
Age	0.11	[0.08, 0.15]	0.000	–0.01	[−0.02, 0.04]	0.489	–0.01	[−0.02, 0.04]	0.550	0.01	[−0.02, 0.04]	0.589
HISEI	–0.12	[0.09, 0.16]	0.000	–0.07	[0.04, 0.10]	0.000	–0.07	[0.04, 0.10]	0.000	0.07	[0.04, 0.11]	0.000
Migration Status	–0.19	[−0.23, −0.16]	0.000	–0.09	[−0.12, −0.06]	0.000	–0.09	[−0.12, −0.06]	0.000	–0.09	[−0.12, −0.06]	0.000
BFI-2 Con.	–	–	–	–	–	–	–0.01	[−0.02, 0.04]	0.398	0.03	[−0.01, 0.06]	0.161
TSC	–	–	–	–0.01	[−0.04, 0.02]	0.570	–	–	–	–0.02	[−0.06, 0.01]	0.187
Fluid Int.	–	–	–	–0.58	[0.55, 0.61]	0.000	–0.58	[0.55, 0.61]	0.000	0.58	[0.55, 0.61]	0.000
TSC x Fluid Int.	–	–	–	–0.01	[−0.02, 0.04]	0.453	–	–	–	0.01	[−0.02, 0.04]	0.460
	*R*^2^ = 0.08 (adjusted *R*^2^ = 0.08)			*R*^2^ = 0.37 (adjusted *R*^2^ = 0.37)			*R*^2^ = 0.37 (adjusted *R*^2^ = 0.37)			*R*^2^ = 0.37 (adjusted *R*^2^ = 0.37)		

*M1, only covariates; M2, covariates, IQ, and TSC; M3, covariates, conscientiousness, and IQ; M4, all variables. TSC, trait self-control; HISEI, Highest International Socio-Economic Index of Occupational Status; con., conscientiousness; mGPA, major grade point average; est., estimate; CI, confidence interval; fluid rea., fluid reasoning.*
